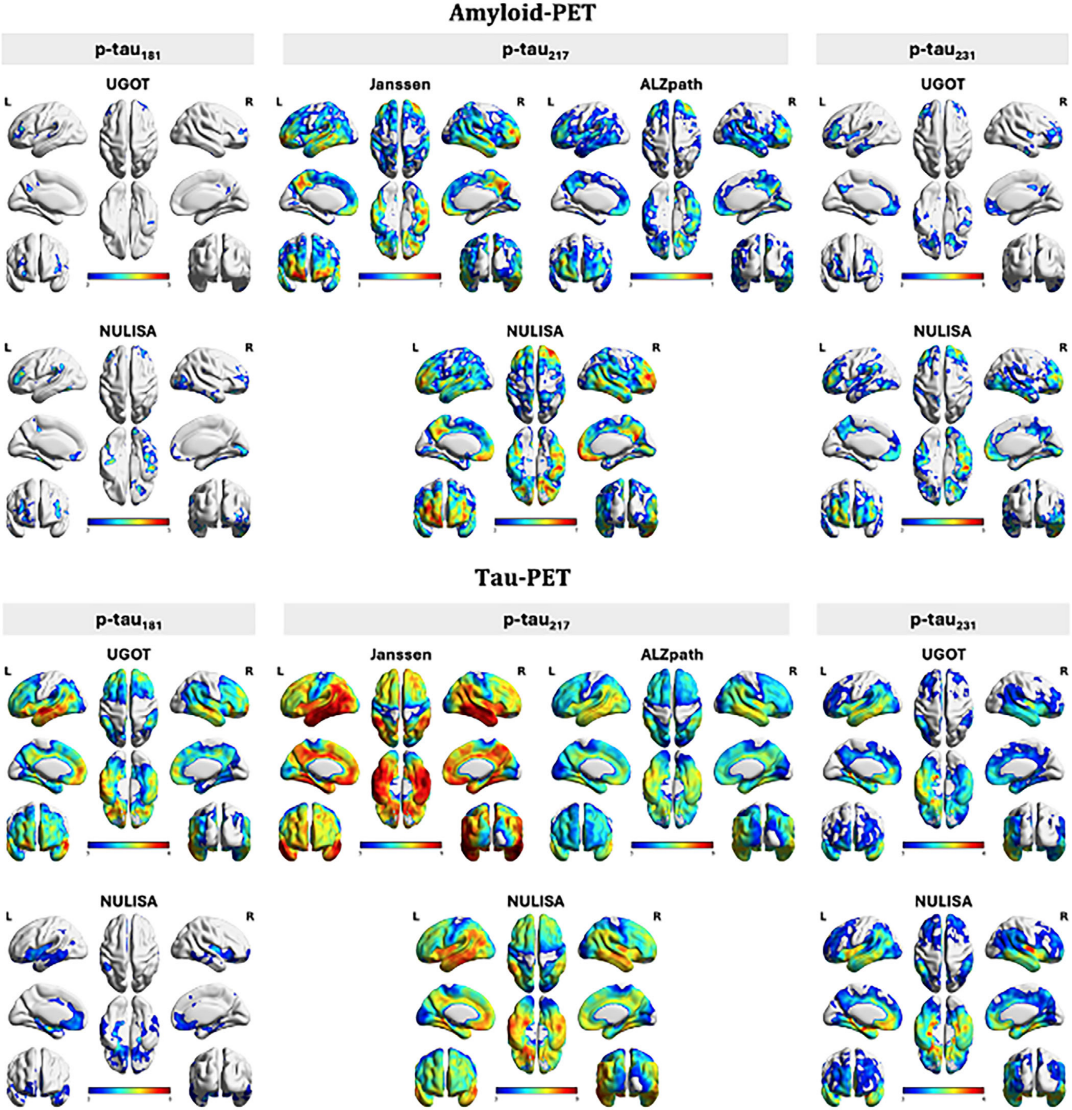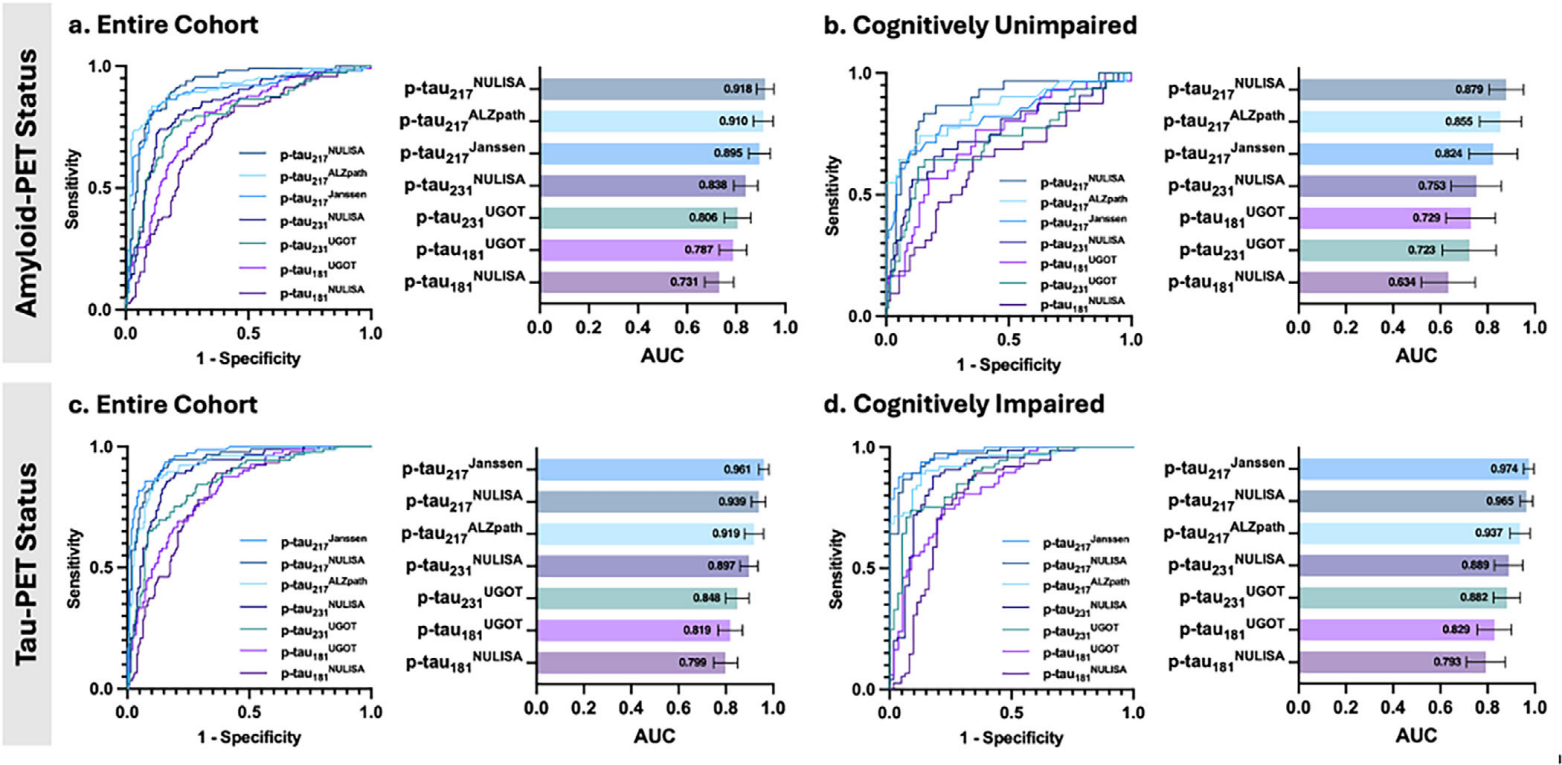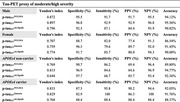# Validation of the plasma phosphorylated tau quantified using NUcleic acid Linked Immuno‐Sandwich Assay (NULISA) for the detection of Alzheimer’s disease pathology

**DOI:** 10.1002/alz.094671

**Published:** 2025-01-09

**Authors:** Yi‐Ting Wang, Nicholas J. Ashton, Joseph Therriault, Andrea L. Benedet, Arthur C. Macedo, Ilaria Pola, Etienne Aumont, Guglielmo Di Molfetta, Jaime Fernandez Arias, Kübra TAN, Nesrine Rahmouni, Stijn Servaes, Richard S. Isaacson, Tevy Chan, Seyyed Ali Hosseini, Cécile Tissot, Sulantha Mathotaarachchi, Jenna Stevenson, Firoza Z Lussier, Tharick Ali Pascoal, Serge Gauthier, Kaj Blennow, Henrik Zetterberg, Pedro Rosa‐Neto

**Affiliations:** ^1^ Translational Neuroimaging Laboratory, The McGill University Research Centre for Studies in Aging, Montréal, QC Canada; ^2^ University of Gothenburg, Mölndal, Gothenburg Sweden; ^3^ Department of Psychiatry and Neurochemistry, Institute of Neuroscience and Physiology, The Sahlgrenska Academy, University of Gothenburg, Mölndal, Gothenburg Sweden; ^4^ Department of Psychiatry and Neurochemistry, Institute of Neuroscience and Physiology, The Sahlgrenska Academy, University of Gothenburg, Mölndal Sweden; ^5^ Institute for Neurodegenerative Diseases (IND) Florida, Boca Raton, FL USA; ^6^ Lawrence Berkeley National Laboratory, Berkeley, CA USA; ^7^ University of Pittsburgh, Pittsburgh, PA USA; ^8^ McGill University, Montreal, QC Canada

## Abstract

**Background:**

Blood‐based biomarkers have been revolutionizing the detection, diagnosis and screening of Alzheimer’s disease (AD). Antibody‐based immunoassays are powerful tools to investigate pathological changes indicated by blood‐based biomarkers and have been studied extensively in AD research. A novel proteomic technology ‐ NUcleic acid Linked Immuno‐Sandwich Assay (NULISA) – was developed to improve the sensitivity of traditional proximity ligation assays and offer a comprehensive outlook for protein biomarkers in neurodegenerative diseases. Due to the relative novelty of the NULISA technology in quantifying AD plasma biomarkers, validation through comparisons with more established methods is required.

**Method:**

In this present study, we assessed 397 participants from the Translational Biomarkers in Aging and Dementia (TRIAD) cohort where participants had plasma measurements of p‐tau_181_, p‐tau_217_ and p‐tau_231_ from both NULISA and other established immunoassays. Participants also underwent neuroimaging assessments including MRI, amyloid and tau positron emission tomography (PET).

**Result:**

Our findings suggest an excellent agreement between plasma p‐tau variants quantified using different immunoassays and strong associations with PET signals in the brain (Fig. 1). As shown in Figure 2, similar to p‐tau_217_ quantified using Janssen and ALZpath immunoassays, plasma p‐tau_217_^NULISA^ shows excellent discriminative accuracy for abnormal amyloid‐PET (AUC = 0.918, 95%CI: 0.883 to 0.953, P < 0.0001) and abnormal tau‐PET status (AUC = 0.939; 95%CI: 0.909 to 0.969, P < 0.0001). It also presents the capability for differentiating tau‐PET staging (Table 1).

**Conclusion:**

Validation of the NULISA CNS panel adds to the current analytical methods for AD diagnosis, screening, and staging, and could potentially expedite the development of a blood‐based biomarker panel.